# Exhaled volatile organic compounds for phenotyping chronic obstructive pulmonary disease: a cross-sectional study

**DOI:** 10.1186/1465-9921-13-72

**Published:** 2012-08-23

**Authors:** Maria Basanta, Baharudin Ibrahim, Rachel Dockry, David Douce, Mike Morris, Dave Singh, Ashley Woodcock, Stephen J Fowler

**Affiliations:** 1 Respiratory Research Group, Faculty of Medical and Human Sciences, University of Manchester, Manchester Academic Health Science Centre, and NIHR Translational Research Facility in Respiratory Medicine, University Hospital of South Manchester, Southmoor Road, Manchester M23 9LT, UK; 2 Waters Corporation, Manchester, UK; 3 Lancashire Teaching Hospitals NHS Foundation Trust, Preston, UK

**Keywords:** Chronic obstructive pulmonary disease, Biomarkers, Breath tests, Metabolomics

## Abstract

**Background:**

Non-invasive phenotyping of chronic respiratory diseases would be highly beneficial in the personalised medicine of the future. Volatile organic compounds can be measured in the exhaled breath and may be produced or altered by disease processes. We investigated whether distinct patterns of these compounds were present in chronic obstructive pulmonary disease (COPD) and clinically relevant disease phenotypes.

**Methods:**

Breath samples from 39 COPD subjects and 32 healthy controls were collected and analysed using gas chromatography time-of-flight mass spectrometry. Subjects with COPD also underwent sputum induction. Discriminatory compounds were identified by univariate logistic regression followed by multivariate analysis: 1. principal component analysis; 2. multivariate logistic regression; 3. receiver operating characteristic (ROC) analysis.

**Results:**

Comparing COPD *versus* healthy controls, principal component analysis clustered the 20 best-discriminating compounds into four components explaining 71% of the variance. Multivariate logistic regression constructed an optimised model using two components with an accuracy of 69%. The model had 85% sensitivity, 50% specificity and ROC area under the curve of 0.74. Analysis of COPD subgroups showed the method could classify COPD subjects with far greater accuracy. Models were constructed which classified subjects with ≥2% sputum eosinophilia with ROC area under the curve of 0.94 and those having frequent exacerbations 0.95. Potential biomarkers correlated to clinical variables were identified in each subgroup.

**Conclusion:**

The exhaled breath volatile organic compound profile discriminated between COPD and healthy controls and identified clinically relevant COPD subgroups. If these findings are validated in prospective cohorts, they may have diagnostic and management value in this disease.

## Background

Chronic obstructive pulmonary disease (COPD) is defined clinically on the basis of airflow obstruction [1,2]. This simple definition does not reflect the heterogeneous nature of COPD with individual variations in pathophysiology, aetiology, symptoms, prognosis and treatment response. For example, chronic cough and mucus are poor prognostic features [3], frequent exacerbations are associated with progressive loss in lung function [4], and the presence of sputum eosinophilia predicts response to inhaled corticosteroids [5]. Identifying these and other subtypes will be critical for appropriate management of these patients in the future, allowing targeted therapy and more accurate disease monitoring [6].

The breath contains as yet unknown numbers of volatile organic compounds (VOCs), both exogenous and endogenous in origin. Endogenous VOCs will arise not only from all levels of the airway but also the circulation via the alveolar-capillary interface, as a result of metabolic processes occurring both in health and disease. Exogenous VOCs can be altered by processes within the airways, both physical (e.g. adsorption into surface liquid) and biochemical (e.g. oxidation). Controlled measurement of these exhaled compounds could therefore potentially give us novel insights into airway biology, physiology and pharmacology, and provide us with phenotype-specific biomarkers. Previous work has suggested that patterns of VOCs in breath gas may be useful in identifying patients with COPD [7], [8], and that individual compounds correlate with inflammatory cell numbers and markers of activation [9], whilst another method for metabolomic analysis has employed nuclear magnetic resonance spectroscopy of exhaled breath condensate to classify COPD and healthy controls [10]. To our knowledge the possibility of identifying clinically relevant disease phenotypes, such the eosinophilic or exacerbation-prone phenotypes, has not been investigated.

We have developed a method that enables us to non-invasively sample the late-expiratory breath in patients with respiratory disease, and used it to discriminate similarly relevant phenotypes in asthma [11]. To ensure detection of potential compounds of interest present at extremely low concentration we have allied our sampling methodology (which concentrates a high volume of breath) to highly sensitive separation and detection instrumentation [gas chromatography-time-of-flight mass spectrometry (GC-ToF-MS)]. The aim of the current study was to use this to characterise the exhaled VOC profile in patients with COPD in comparison to healthy controls. We have then investigated whether VOC profiles can discriminate subgroups defined by inflammatory-cell phenotype and exacerbation frequency.

## Methods

### Subjects

Subjects were recruited from the Medicines Evaluation Unit clinical trials database, University Hospital of South Manchester, UK. Some of the COPD subjects were also taking part in the Evaluation of COPD Longitudinally to Identify Predictive Surrogate End-points (ECLIPSE) study [12]. The study was approved by a local Research Ethics Committee and subjects provided written informed consent. All COPD subjects had a clinical diagnosis of the disease, and met Global Initiative for Chronic Obstructive Lung Disease (GOLD) criteria [1].

### Inclusion and exclusion criteria

Participants with COPD had baseline post-bronchodilator forced expiratory volume in one second (FEV1)/forced vital capacity (FVC) less than 0.7 and a smoking history of at least 10 pack years. Healthy volunteers had baseline FEV1 greater than 80% predicted, FEV1/FVC greater than 0.7, and were categorised as healthy smokers (current smokers with at least 10 pack year history) or healthy never-smokers. Abstinence from smoking was checked by measurement of exhaled carbon monoxide with a cut-off of ≤5 ppm (Smokerlyzer, Bedfont Scientific, Maidstone, UK). Exclusion criteria included: other chronic respiratory disorders or systemic inflammatory disease, (e.g. rheumatoid arthritis), malignancy within last five years, moderate or severe exacerbation (requiring oral corticosteroids and/or antibiotics) within the four weeks prior to study visit (all courses of oral corticosteroids and antibiotics must have been completed at least 2 weeks before study visit), long term (more than three months) oral corticosteroids.

### Study procedures

Subjects were asked to refrain from eating, drinking (except for water) and smoking for two hours prior to breath collection, and from using their inhaled medication on the morning of the visit. Demographic data were collected, and subjects from the ECLIPSE cohort had prospectively recorded exacerbation frequency in the previous year. Study procedures were then performed in the following order: exhaled breath collection, spirometry, and sputum induction.

### Spirometry

Spirometry was performed according to American Thoracic Society / European Respiratory Society guidelines [13], using the MasterScope CT pneumotach (Viasys GmbH, Hoechberg, Germany).

### Sputum induction

Sputum induction was performed using inhaled hypertonic saline in increasing strengths (3, 4, and 5%) from an ultrasonic nebuliser (Medix Sonix 2000, Clement Clarke, Essex, UK) as previously described [14]*.* Sputum induction was stopped early if the patient’s FEV_1_ fell below the safety cut-off (80% of baseline). Sputum samples were stored on ice and processed within two hours. A minimum of 400 leukocytes were counted. Slides containing > 20% squamous cells were regarded as representing salivary contamination and excluded.

### Exhaled breath collection and analysis

All the breath samples were collected in the same room, used solely for this study, minimising the effect of variation in background air. Breath samples were collected and analysed as previously described [11,15]. In brief subjects breathed VOC-filtered air, while respiratory pattern was tracked via a pressure transducer and visualised using bespoke software, enabling selective sampling of late expiratory breath, minimising contamination from the mouth, nose and deadspace. Sampling was performed during tidal breathing, and commenced after the subject had been breathing VOC filtered air for five minutes, allowing a degree of equilibration as well as ensuring the subjects were relaxed at the start of sampling. Three litres of selected exhaled breath per sample were collected directly onto adsorbent tubes packed with Tenax TA/Carbotrap (Markes International, Rhondda Cynon Taff, UK) for analysis by GC-ToF-MS. Due to differing tidal volumes between subjects, and with sampling triggered only during late expiration, collection of each 3 L sample typically took between five and seven minutes.

### Sample analysis and data processing

Samples were analysed in random order by thermal desorption (TD) followed by GC-ToF-MS. To ensure the instrument response was precise for the wide range of VOCs that we expected to detect, a quality control (QC) was made up of a mixture of 21 VOCs (Sigma-Aldrich; purity 99%; solvent HPLC grade methanol) and run through the instrument before each study sample. A range of concentrations from pg/μl to ng/μl were prepared to calibrate the instrument. D5-bromobenzene was added to the breath samples and QC as an internal standard prior to analysis at a 1.5 ng/μl concentration (RSD 5.14%). The analytical methodology, including details of the QC and internal standard, is described elsewhere [15]. Instrumental and intra-individual (day-to-day) reproducibility have previously been shown to be excellent for breath samples analysed by GC-ToF-MS [16].

Data were acquired and pre-processed using the on-board software package MassLynx (Waters Corp., Manchester UK). Pre-processing entails detection, spectral deconvolution and alignment of potential markers. Markers were presented as exact mass and retention time pairs and the intensity of each marker for each sample was recorded. Principal component analysis (PCA) was performed on the marker intensities to visualise any major differences between sample sets. In parallel Automated Mass Spectral Deconvolution and Identification System (AMDIS) was used to extract spectra for individual components from GC-MS data and identifies compounds by matching these spectra against specialised libraries. A reference file containing straight chain alkanes was used to build up a retention indices library to allow alignment of data, and a library of 487 compounds created by confirming the identity of compounds against the National Institute of Standards and Technology (NIST) library and elemental composition of molecular ions and their fragments. Absolute error was acceptable at values lower than 1.5 mDa. Internal standard peak area was extracted and relative intensities of library compounds calculated (i.e. normalised to the internal standard). Statistical analysis was performed on a final data matrix containing relative peak areas of library components for all the samples.

### Statistical analysis

All data were analysed using SPSS version 15 (SPSS Inc., Chicago, IL, USA). For demographic data, descriptive statistics were used, with between group comparisons made using Pearson chi square, parametric (students t-test) and non parametric tests (Mann Whitney U) where appropriate.

**Figure 1:**
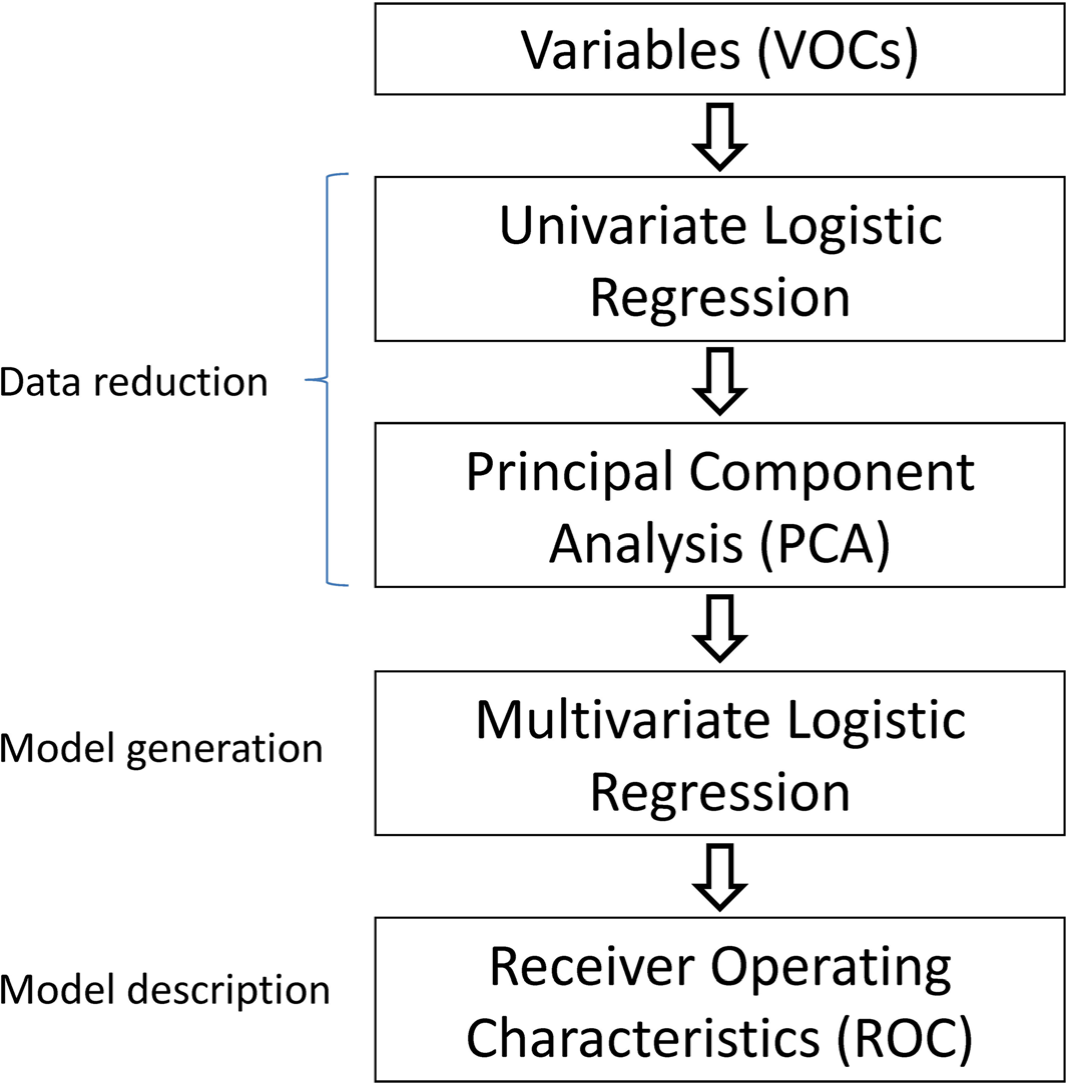
Flow-chart summarising the statistical approach adopted for variable reduction and model generation.

The primary comparison of interest was the breath profile of COPD patients *versus* healthy controls. It was not possible to calculate a sample size for this study as we had no *a priori* information regarding the identity or variance of potential compounds of interest. We anticipated that smoking status and inhaled corticosteroid use would represent obvious confounders to the analysis, so planned to stratify by current smoking status, and COPD subgroup analysis by inhaled corticosteroid use. To investigate the potential for the breath profile to predict clinical phenotypes, we planned further COPD subgroup analyses by sputum eosinophilia and exacerbation frequency. Cut-offs for sputum eosinophilia and exacerbation frequency were pragmatic: a cut-off for sputum eosinophils of 1% can identify non-responders to inhaled corticosteroids in COPD [5] whereas higher cut-offs such as 2% are typically used in severe asthma [17]; there are no similar data to guide a cut-off for exacerbation frequency, so we used the median value in our cohort of two exacerbations in the previous 12 months. The aim of the data analysis was to generate a valid model for discriminating between groups. Our analytical strategy has previously been published [11], and is summarised in Figure 1. One approach in metabolomics for dealing with the very high number of variables in comparison to the number of subjects is to perform initial data reduction prior to multivariate analysis (e.g. [18,19]). For each comparison of interest this was achieved by between group univariate analysis (logistic regression) for each of the identified compounds, in order to achieve a balance between optimising the validity of the subsequent principal component analysis (PCA), whilst limiting the risk of discarding potentially important compounds. The principal components (PCs) were then entered into multivariate logistic regression to generate a best-fit model for between group discrimination, and the performance of this model described by receiver operating characteristics [20]. Discriminant function analysis (DFA) with leave-one-out cross validation (LOOCV) was used in parallel to check the performance of the model. The relationship between specific VOCs forming the PCs used in each model and the corresponding phenotype-defining parameters (e.g. sputum eosinophil count, number of exacerbations per year) was explored further using Pearson’s Correlation Coefficient.

## Results

Breath samples were collected from a total of 71 subjects (39 COPD and 32 healthy controls), including 23 COPD subjects recruited from the ECLIPSE cohort. Demographic details are shown in Table 1. Apart from the expected differences in lung function parameters, the COPD group were also older and had more comorbidities than the healthy group. None of the included subjects in either group had a history of renal or hepatic impairment.

**Table 1:** Demographic data for patients with COPD and healthy controls shown as mean (SD) or %

		**COPD (n = 39)**	**Healthy (n = 32)**
Age, yrs		65.7 (6.8)	55.3 (7.1) *
Gender (% male)		67	47
Smoking status (%):	Current	31	31
	Ex	69	0
	Never	0	69
	Pack years	45.6 (18.9)	37.3 (19.1)
FEV_1_, l		1.48 (0.5)	3.26 (0.8) *
FEV_1_,% predicted		54.6 (15.4)	102.4 (12.1) *
FVC, l		3.12 (0.8)	4.20 (0.9) *
FVC,% predicted		89.7 (17.6)	113.3 (14.6) *
FEV_1_ / FVC		49.2 (13.1)	77.5 (6.6) *
BMI, kg / m^2^		27.8 (5.6)	27.4 (3.6)
GOLD class I/II/III/IV (%)		2/19/16/2	NA
Inhaled corticosteroids (%)		64	NA
Dose, μg BDP equivalent		842 (978)	NA
Long acting β_2_-agonists (%)		56	NA
Long acting antimuscarinics (%)		56	NA

* p < 0.05 for COPD versus healthy controls.

GOLD = Global Initiative for Chronic Obstructive Lung Disease [1]; BDP = beclomethasone dipropionate.

### COPD *versus* healthy controls

**Figure 2:**
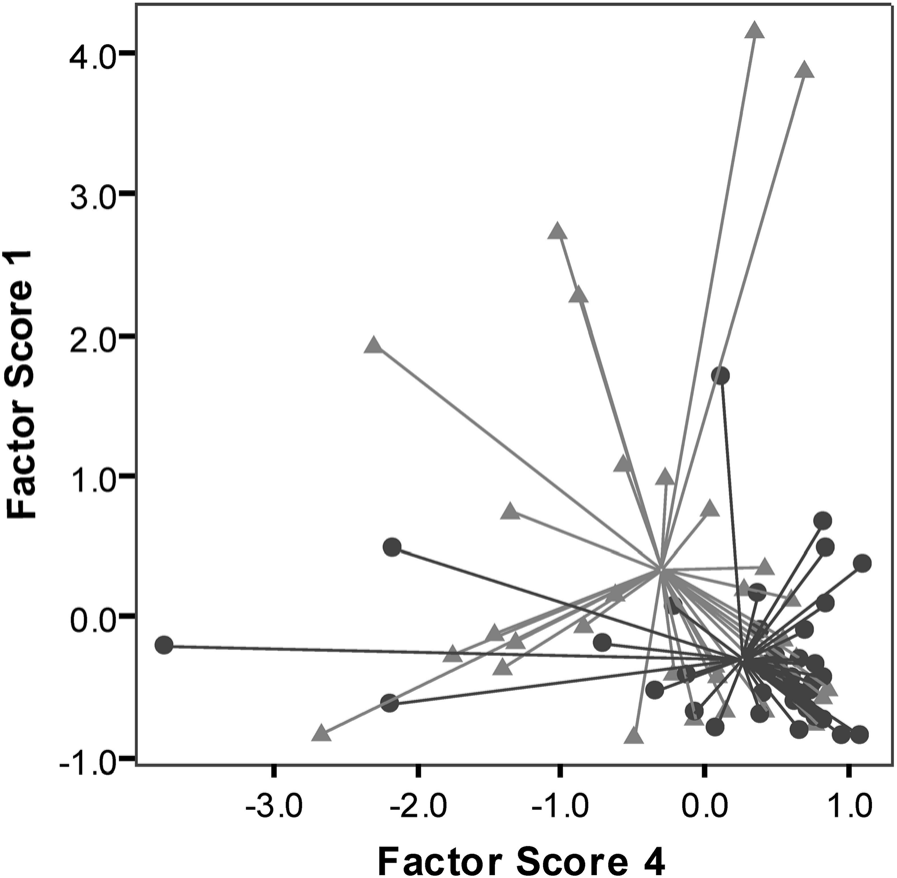
**Scatter plot of the principal components (labelled as “factor scores”) used in the multivariate logistic regression model for discriminating COPD (triangles) *versus *healthy controls (circles). **Each data point is linked to the centroid, demonstrating the central point of each distribution.

From the 487 compounds identified, those with COPD *versus* healthy control p < 0.10 (n = 20) were retained for inclusion in the PCA. Four PC’s with Eigenvalues more than one and explaining 70.8% of the total variance were derived. When these four PC’s were used in logistic regression analysis, a model consisting of PC 1 and PC 4 significantly predicted samples from subjects with COPD. This model is shown in Table 2, and correctly classified 33 (84.6%) of COPD samples and 16 healthy controls (50.0%). A plot of the PCs used in the model is shown in Figure 2. The ROC parameters for the model were: sensitivity 85%, specificity 50%, precision 67%, accuracy 69%, area under ROC curve (AUROC) 0.74. These results were confirmed with discriminant function analysis which showed the two factors significantly discriminated the two groups with accuracy of 70.4% [Wilk’s lambda = 0.84, chi square = 11.909, p = 0.003], and this accuracy was confirmed using LOOCV which also showed accuracy of 70%. A list of the putative compounds (six of which were aldehydes) contributing to the discriminatory model is shown in Table 3.

**Table 2:** **Logistic regression model for COPD*versus*healthy controls**

	**B (SE)**	**P value**	**Odds ratio**	**95% Confidence interval for odds ratio**
PC 1	−0.751 (0.366)	0.040	0.472	0.230 to 0.967
PC 4	0.549 (0.284)	0.054	1.731	0.992 to 3.022
Constant	0.159 (0.265)			

**Table 3:** Empirical formulae and putative identification of volatile organic compounds used in the model classifying COPD and healthy subjects, grouped by principal component (PC)

**PC**	**Loading**	**Compound ID**
1	.987	Undecanal C_11_H_22_O
	.949	Hexanal C_6_H_12_O
	.901	Dodecanal C_12_H_24_O
	.874	Decanal C_10_H_20_O
	.867	Nonanal C_9_H_18_O
	.764	Pentadecanal- C_15_H_30_O_2_
	.741	Oxirane, dodecyl- C_14_H_28_O
	.740	C_8_H_14_O_3_ Cyclohexanol, 5-methyl-2-(1-methylethyl)-, [1R-(1à,2á,5à)]- C_10_H_20_O
4	-.829	Butanoic acid, 2,2-dimethyl-3-oxo-, ethyl ester C_8_H_14_O_3_
	-.710	Pentanoic ac C_5_H_10_O_2_
	-.498	Furan,2-pentyl C_9_H_14_O

Also shown are PC loadings; loadings closer to one indicate compounds with a higher influence in the PC.

We speculated that this relatively poor discrimination may have been due to the dominating effect of cigarette smoke-related VOCs in some samples, and therefore studied subgroup comparisons based on smoking status. When comparing ex-smokers with COPD with healthy non-smokers the multivariate model accuracy was 73%, and ROC parameters: sensitivity 89%, specificity 55% and AUROC 0.77. When data from current smokers only were examined, the resultant model had accuracy 91% and ROC sensitivity 92%, specificity 90% and AUROC 0.98.

We investigated whether any of the discriminatory compounds were likely to arise from a corticosteroid effect in the COPD group. Multivariate logistic regression generated a model which discriminated the COPD subjects taking inhaled steroid (n = 25) from those not on inhaled steroid (n = 14) with accuracy of 74% and AUROC of 0.83. Of the discriminatory compounds included in the COPD *versus* healthy model (Table 3), only undecanal was also found amongst the compounds included in the steroid model.

### Clinical subgroup analysis

Demographic details of the subgroups are shown in Table 4.

a) Sputum eosinophilia

**Figure 3:**
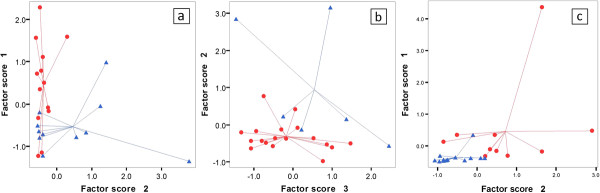
**Scatterplots of the principal components derived from the models predicting (blue triangles): a. sputum eosinophils ≥ 1%; b. sputum eosinophils ≥ 2%; c. ≥ 2 exacerbations per year. **Figure 3**b** for example shows that plotting factor scores 1 *versus* 2 from the model generated to discriminate based on sputum eosinophils ≥ 2% enables clear separation of the groups, with only two of the eosinophilics, and none of the non-eosinophilics, being misclassified.

**Figure 4:**
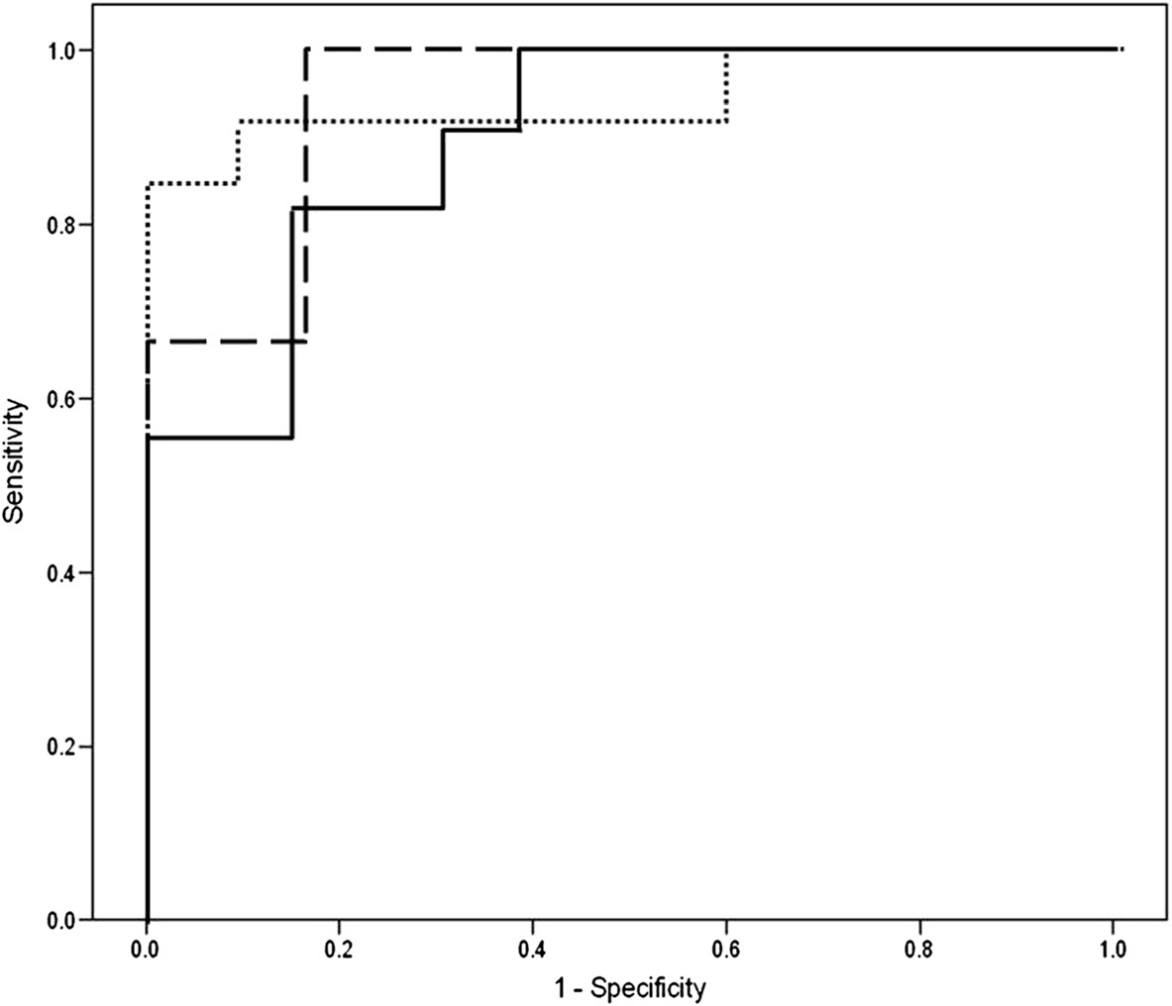
Receiver operating characteristics curves for subgroup comparison. Key: solid line - sputum eosinophils ≥1%; dashed line - sputum eosinophils ≥2%; dotted line - exacerbation frequency ≥ 2 / yr.

There were no significant demographic differences between subjects with eosinophilic or non-eosinophilic sputum using either cut-off (1% or 2%) in terms of age, gender or lung function. Eleven of 24 COPD subjects with evaluable sputum samples had eosinophil count ≥ 1%, and six ≥ 2%. The logistic regression models had accuracy of 79% and 92%, AUROC of 0.90 and 0.94, and LOOCV accuracy of 75% and 88% respectively. Plots of the PCs used in the model are shown in Figures 3a and 3b, the ROC curve in Figure 4, and compounds underlying the PC’s in Table 5.

**Table 4:** Demographic details for subgroup comparisons. Data are shown as mean (SD), except for gender

	**Sputum eosinophilia**						**Exacerbation frequency**		
	**≥ 1% (n = 11)**	**< 1% (n = 13)**	**p**	**≥ 2% (n = 6)**	**< 2% (n = 18)**	**p**	**<2 (n = 10)**	**≥2 (n = 13)**	**p**
Age	65.0 (6.7)	64.5 (7.3)	0.884	63.5 (8.3)	65.2 (6.6)	0.713	67.0 (5.2)	66.5 (4.7)	0.686
Gender (male/female)	7/4	9/4	0.772	3/3	13/5	0.317	7/3	9/4	0.968
Cigarette pack years	48.3 (19.8)	39.7 (12.0)	0.199	38.0 (14.1)	45.5 (16.9)	0.338	49.7 (27.9)	49.9 (10.3)	0.983
% predicted FEV_1_	51.5 (16.9)	59.4 (9.3)	0.131	58.2 (19.3)	55.0 (11.7)	0.713	52.9 (11.9)	44.9 (14.3)	0.120
ICS total daily dose (μg BDP equivalent)	570 (797)	818 (756)	0.473	825 (826)	300 (346)	0.058	880 (855)	1364 (1196)	0.400

**Table 5:** Table of putative VOCs and empirical formulae associated with sputum eosinophilia and frequent exacerbations, shown in order of decreasing strength of correlation (r) between compounds and sputum eosinophil count, and number of exacerbations in the previous year

**Eosinophils ≥ 1%**	**r**	**Eosinophils ≥ 2%**	**r**	**Exacerbations ≥ 2 / yr**	**r**
**α-Methylstyrene C**_**9**_**H**_**10**_	−0.51	**1, 1'-Biphenyl, 3-methyl- C**_**13**_**H**_**12**_	−0.49	**Undecane, 3, 7-dimethyl C**_**13**_**H**_**28**_	−0.63
**3-Cyclohexen-1-ol, 4-methyl-1-(1-methylethyl)-, acetate C**_**12**_**H**_**20**_**O**_**2**_	−0.51	dodecanoic ac methylethylester C_15_H_30_O_2_	0.31	**2, 2, 4, 4-Tetramethyloctane C**_**12**_**H**_**26**_	−0.58
**benzofuran, 4, 5, 6, 7 tetrahydro-3, 6-dimethyl C**_**10**_**H**_**14**_**O**	0.41	benzene,1, 2, 3, 4-tetramethyl- C_10_H_14_	0.29	**1,4-Methanoazulene, decahydro-4, 8, 8-trimethyl-9-methylene-, [1S-(1α,3aβ,4α,8aβ)]- C**_**15**_**H**_**24**_	−0.53
**Decane, 3-methyl C**_**11**_**H**_**24**_	0.41	bicycle carene type C_10_H_16_	0.23	**Naphthalene, 2, 3, 6-trimethyl- C**_**13**_**H**_**14**_	−0.49
Pentanoic acid, 2, 2, 4-trimethyl-3-carboxyisopropyl, isobutyl ester C_16_H_30_O_4_	−0.33	1, 5-Heptadiene, 2, 5-dimethyl-3-methylene- C_10_H_16_	0.20	Chlorobenzene H_6_H_5_Cl	−0.37
2 cyclopenten1one 3, 5, 5 trimethyl C_8_H_12_O	−0.31			Pentadecane, 3-methyl- C_16_H_34_	−0.37
2(1 H)Naphthalenone, 3, 5, 6, 7, 8, 8a-hexahydro-4, 8a-dimethyl-6-(1-methylethenyl)- C_15_H_22_O	0.30				

Compounds with significant r values (p ≤ 0.05) shown in bold.

b) Exacerbation frequency

Data on the number of exacerbations in the previous 12 months were available for the 23 COPD subjects recruited from the ECLIPSE cohort. Thirteen had had two or more exacerbations in that time. Logistic regression predicted the group with frequent exacerbations with an accuracy of 87%, area under ROC curve 0.95, and LOOCV accuracy of 83%. A plot of the PC’s is shown in Figure 3c, the ROC curve in Figure 4, and relevant compounds in Table 5.

## Discussion

We have shown that this technique for breath collection paired with metabolomic VOC analysis by GC-ToF-MS is able to classify COPD from healthy controls with moderate accuracy. However we were able to demonstrate much greater accuracy when looking at sub-groups of clinical interest such as smokers with COPD *versus* asymptomatic smokers, and COPD subjects with sputum eosinophilia, or those liable to suffer frequent exacerbations. If validated in prospective cohorts, this technique may provide a non-invasive method for phenotyping COPD in the future, with clinical applications for example in personalised therapeutics and prognosis.

Recent data have also shown discrimination between COPD and healthy controls using GC-MS, but based upon analysis of single breath samples [7]. Current work in metabolomic analysis of exhaled VOCs is exploratory and aimed at discovering potential novel biomarkers, so it is critical that the sensitivity of the detection system be optimised. Each of our breath samples contains the VOCs absorbed from 3 litres of late-expiratory air, typically representing 50 to 100 breaths per subject. Paired with GC-ToF-MS, we have a highly sensitive methodology for both absorption and detection of VOCs that may well be present in minute concentrations in a single breath. It is of interest that the classification model developed in the Van Berkel study had higher accuracy for discriminating COPD *versus* controls than ours. Although the breath collection methodologies differed, the analytical techniques, based on GC-ToF-MS, were similar. It may be that the large difference in demographics between the COPD and healthy cohorts in that study (the COPD group were 21 years older, and 76% were current smokers) contributed to the differences seen. Likewise the close matching of current smoking status in our cohorts may have contributed to the moderate success of our classification model, as active smoking is clearly likely to be a dominant confounder in exhaled breath analysis where data from both smokers and non-smokers are analysed together. Indeed when we looked only at smokers with and without COPD (thus neutralising this confounding effect in the analysis), we found our model classified disease with far greater accuracy. The scatter plot (Figure 2) shows the COPD data to be clustered in one region of (but still within) the data from the healthy controls. This is consistent with the concept of COPD as a disease of accelerated lung aging [21], and it is perhaps not surprising that the metabolomic profile of patients with this disease clusters at one extreme of normality, rather than apart from it.

Sputum eosinophilia predicts steroid-responsiveness in COPD [5], but the test is labour-intensive, time consuming, and samples are not obtainable in a significant minority of patients [22]. Whilst our technique for breath collection and analysis is currently relatively high-cost and labour-intensive, if a set of candidate biomarkers were validated for predicting steroid responsiveness, work would focus on developing and producing small, user-friendly point-of-care sensors specifically for this purpose. We have previously shown that breath VOC profiles can also predict sputum eosinophilia in asthma [11]. Unsurprisingly, given the differences in demographics and disease processes between the studies, the specific VOCs used in the models were not the same. The identification of VOCs patterns specific to sputum inflammatory profile, and phenotypes such as “frequent exacerbators” may not only provide biomarkers for clinical use, but also could potentially provide new insights in disease pathophysiology.

Whilst absolute control of environmental VOCs is practically impossible, it is desirable to reduce background levels as much as is practicable. We use a VOC-filter in our circuit, an equilibration time of at least five minutes, and collect samples in the same room for this purpose. Even so, exogenous VOCs may be differentially handled by the airways in health and disease, and alteration in exhaled concentrations may therefore be relevant. Examples include: low-molecular weight molecules being absorbed by excessive airway secretions more readily than heavier molecules; the systemic circulation, acting as a reservoir, re-releasing environmental VOCs into the air at rates determined by cardiac output and lung circulation [23]; airway and alveolar inflammatory processes metabolising inhaled VOCs for example by oxidation; and the air trapping seen in obstructive lung disease altering the washout time for gas-phase molecules compared to healthy lungs. One approach to correcting for “exogenous” VOCs is to subtract the content of a contemporaneous environmental sample from the expired sample [24], but this oversimplifies the metabolic and physiological impact of the airways and circulation.

Our findings require validation in an independent group of subjects then definitive compound identification and calibration curves determined by injection of known standards. The origins of these compounds are as yet unknown, but hypotheses can be generated. For example six of the 11 compounds discriminating COPD from health were aldehydes, and all had strong loading onto the first principal component, an interesting finding in line with the findings of Van Berkel *et al.*[7]. It may be that the metabolic upregulation in the mucosa of COPD patients removes aldehydes from the air; it is known for example that the aldehyde scavengers N-acetylcysteine and glutathione monoethyl ester completely remove unsaturated (but not saturated) aldehydes from a cigarette smoke extract [25]. There may also be an effect of ICS on suppressing exhaled aldehyde levels, supported by the contribution of undecanal to both models. Further, it may be instructive in future studies to compare these putative exhaled markers of inflammation to existing disease-relevant breath biomarkers such as leukotriene B_4_ and other eicosanoids [26].

## Conclusion

We have demonstrated the potential of breath gas analysis for the identification of metabolomic patterns that not only can be used to discriminate health from disease (especially amongst current smokers) but also to identify clinically relevant disease phenotypes. It is now essential that these findings be validated prospectively in an independent group of patients in order to confirm that these patterns have potential for clinical use as biomarkers. Furthermore, confirmation of the identity of specific discriminatory compounds may lead to the elucidation of metabolic pathways with potential benefits for novel therapeutic targets.

## Abbreviations

AUC: Area under the curve; COPD: Chronic obstructive pulmonary disease; DFA: Discriminant function analysis; FEV1: Forced expiratory volume in one second; FVC: Forced vital capacity; GC: Gas chromatography; ICS: Inhaled corticosteroids; MS: Mass spectrometry; PC: Principal component; PCA: Principal component analysis; QC: Quality control; ROC: Receiver operating characteristics; RSD: Relative standard deviation; ToF: Time of flight; VOCs: Volatile organic compounds.

## Competing interests

The authors declare that they have no competing interests.

## Authors’ contributions

MB, BI and RD were involved in performing study procedures, data collection and analysis; DD and MM provided technical supervision and advice; DS and AW provided clinical supervision and advice and SJF provided overall project supervision. All authors were involved in study design and had input into and approved the final manuscript.
